# Unravelling the Extent of Diversity within the Iberian Medicinal Leeches (Hirudinea: *Hirudo*) Using Molecules and Morphology

**DOI:** 10.3390/biology10040315

**Published:** 2021-04-09

**Authors:** Andrés Arias, Victor Surugiu, Rafael Carballeira, Oana Paula Popa, Luis Ovidiu Popa, Serge Utevsky

**Affiliations:** 1 Departamento de Biología de Organismos y Sistemas (Zoología), Universidad de Oviedo, 33071 Oviedo, Spain; 2 Faculty of Biology, “Alexandru Ioan Cuza” University of Iasi, 700505 Iaşi, Romania; vsurugiu@uaic.ro; 3 Centro de Investigacións Cientificas Avanzadas (CICA), Facultade de Ciencias, Universidade da Coruña, 15071 A Coruña, Spain; rafael.carballeira@gmail.com; 4 Grigore Antipa National Museum of Natural History, Sos. Kiseleff Nr. 1, 011341 Bucharest, Romania; oppopa@antipa.ro (O.P.P.); popaluis@antipa.ro (L.O.P.); 5 Department of Zoology and Animal Ecology, V. N. Karazin Kharkiv National University, Maidan Svobody 4, 61022 Kharkiv, Ukraine; serge.utevsky@karazin.ua

**Keywords:** Annelida, biodiversity, speciation, COI mtDNA, 12S rRNA, ITS2, conservation, ESU, Europe, taxonomy

## Abstract

**Simple Summary:**

During the last decade, our understanding of the biogeography of Western-Palaearctic leeches (genus *Hirudo*) has begun to unravel, unveiling their diversity in practically all of Europe, except for its westernmost peninsula, Iberia. We discovered for the first time *H. verbana* in Spain and conducted an integrative approach (combining morphology, anatomy, ecology with genetics) to characterize the newly recorded Iberian populations. We found two endemic and geographically separated Iberian lineages of *H. verbana*. One of them is easily distinguished by its distinctive colour-pattern and is described as *H. verbana bilineata* ssp. nov. We established its phylogenetic relationships with other European *Hirudo* spp. and confirmed the presence of a second species in Iberia, *H. troctina*. The provided distribution pattern of medicinal leeches contributes to a better understanding of the complexity of the Iberian Peninsula as a glacial refugium/cradle for endemisms, sheltering species populations that began to settle throughout the Pleistocene. Finally, we highlight the urgent need for implementing conservation measures to reverse the evident decline of Iberian populations of medicinal leeches.

**Abstract:**

Until the beginning of the 21st century, the famous medicinal leech was thought to be represented by only one species, *Hirudo medicinalis*. However, recent publications have demonstrated that under that name, at least five different species of medicinal leeches were hidden. During the last decade, the biogeography of Western-Palaearctic leeches has begun to unravel, untangling their diversity in practically all of Europe, except for its westernmost peninsula, Iberia. *Hirudo medicinalis* has been repeatedly reported from Iberia, but those records were considered questionable. We discovered *H. verbana* in northern Spain, constituting its first record in Iberia. Using an integrative approach (combining morpho-anatomical data and molecular analyses using three genes, COI,12S rRNA, and ITS2), two endemic and geographically separated Iberian lineages have been found. One of them is easily distinguished by its distinctive colour-pattern and is described as *H. verbana bilineata* ssp. nov. We characterized the new subspecies morphologically, ecologically, and genetically. We also established its phylogenetic relationships with other European *Hirudo* spp. and confirm the presence of *H. troctina* in Iberia, occurring as far as 43° lat. N. Iberian *H. verbana* records constitute its westernmost known distribution to date. The provided distribution pattern of *H. verbana* contributes to a better understanding of the complexity of Iberia as a glacial refugium/cradle for endemisms, harbouring populations with a high degree of genetic structure that began to settle throughout the Pleistocene. Iberian *Hirudo* populations are declining in recent decades and there is an urgent need to assess their conservation status and to initiate conservation measures to reverse their decline.

## 1. Introduction

The medicinal leech *sensu lato*—*Hirudo medicinalis* Linnaeus, 1758—is one of the first described species of annelids and the most well-known representative of the subclass Hirudinea, which was named and originally defined by this species. Medicinal leeches are haematophagous ectoparasites of vertebrates and are widely used in medicine and as model organism in biology and physiology [1]. *Hirudo medicinalis* has been traditionally considered as a single species; however, several recent publications have demonstrated that what was considered as *H. medicinalis* actually comprises at least five different species: *H. medicinalis*, *Hirudo troctina* Johnson, 1816, *Hirudo verbana* Carena, 1820, *Hirudo orientalis* Utevsky & Trontelj, 2005 and *Hirudo sulukii* Saglam, Saunders, Lang and Shain, 2016 [1,2,3,4,5,6,7]. The biogeography of the Western Palaearctic medicinal leeches has been recently revised by Utevsky et al. [6]. These authors stated that *H. medicinalis* is distributed from Britain and southern Norway to the southern Urals and probably as far as the Altai Mountains; *H. verbana* is distributed from Switzerland and Italy to Turkey and Uzbekistan; *H. orientalis* occurs in the Transcaucasian countries, Iran, and Central Asia; *H. troctina* is present in north-western Africa and southern Iberia [6], and finally *H. sulukii* is, so far, only known from south-eastern Anatolia [7]. Furthermore, the aforementioned authors [6] also concluded that climate conditions play an important role in the distribution patterns of the medicinal leeches in the Western Palaearctic region.

The Iberian Peninsula or Iberia is the third largest European peninsula and constitutes the south-westernmost part of Europe. It is bordered on the southeast and east by the Mediterranean Sea and on the north, west, and southwest by the Atlantic Ocean. The Pyrenees Mountains are situated along the northeast edge of Iberia, where it adjoins the rest of Europe. Its southern tip is very close to the northwest coast of Africa. Due to these geographical features, Iberia harbours different climate types, outstanding the Oceanic climate from the North to north-western part and the Mediterranean one in the Centre and southern regions. It also presents both the Alpine and the Subarctic climate in the higher mountains of northern Spain (mainly the Cantabrian Mountains and the Pyrenees) [8]. From the biogeographical perspective, the Iberian Peninsula is considered one of the most important Pleistocene glacial refugia of the European subcontinent [9,10,11]. Iberia harbours a remarkable biological diversity, including endemic ectoparasites [12].

Two species of medicinal leeches have been repeatedly reported from Iberia: *H. medicinalis*, mostly in the northern part [6,13,14,15,16,17], and *H. troctina*, mostly in the southern Iberian Peninsula [18,19,20,21,22]. However, based upon current knowledge on the taxonomy and biogeography of the European medicinal leeches, the Iberian records of *H. medicinalis* should be considered as questionable [6]. Furthermore, the climate conditions of North-West and North Iberia might favour the occurrence of *H. verbana* in this area, as has been previously hypothesized by Utevsky et al. [6].

During the course of our investigations, we discovered two populations of *Hirudo* from north-western and north-central Spain, characterized by a pigmentation pattern distinct from the typical European *Hirudo* spp. (i.e., *H. medicinalis*, *H. verbana* and *H. troctina*), with a ventral pattern closely resembling of *H. verbana* and a distinctive dorsal pattern, consisting of two paired lines that run parallel at the lateral margins of the dorsum along the entire length of the leech (herein referred to as “bilineated”). Furthermore, we also found several populations bearing the typical colour pattern assigned to *H. verbana* in Galicia (northwestern Spain).

The main goal of the present paper is to assess the actual diversity and distribution of the Iberian medicinal leeches, in order to fill the knowledge gap on the biogeography of south European *Hirudo* spp. This study aims as well to reveal the phylogenetic relationships between the Iberian and the other European medicinal leeches by using two mitochondrial genes and various morpho-anatomical characters.

## 2. Materials and Methods

### 2.1. Species Sampling and Morphological Examination

**Figure 1:**
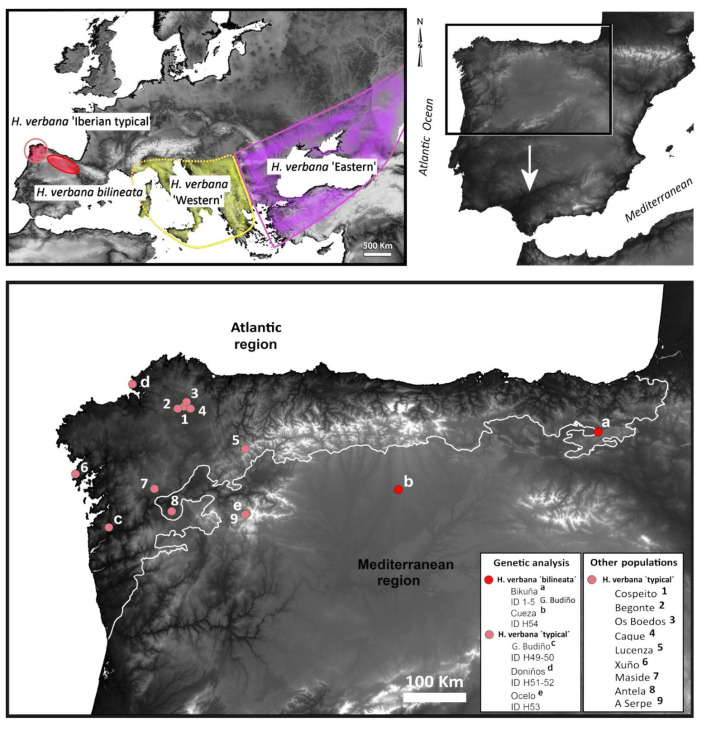
Geographical distribution of *Hirudo verbana* in Europe (top left corner) and in Iberian Peninsula, showing genetic-studied populations (a–e) and others (1–9) (bottom) [Elevation map SRTM 90 m].

The search for *Hirudo* populations in the field took place from May 2015 to October 2017. Successful collections of *Hirudo* spp. were carried out from August 2015 to July 2016 at several locations in the northern Iberian Peninsula (Figure 1). The leeches were prepared for preservation in situ, first relaxed in an ascending series of graded ethanol and afterwards fixed in 10% formalin for morphological studies and in 100% ethanol for genetic studies. Thereafter, collected specimens were brought to the laboratory for subsequent taxonomical and genetic analysis. All localities with collection data and additional information are fully listed in Table 1. Initially the specimens were sorted into two groups or morphotypes according to their colour pattern: “typical”—the originally described pattern in *H. verbana*, and “bilineated”—dorsal colouration consisting of two paired lines that run parallel at the lateral margins of dorsum, along the entire length of the leech.

Additionally, historical collections of *Hirudo* sp. specimens from different localities of the Iberian Peninsula, deposited in the Museo Nacional de Ciencias Naturales (MNCN) of Madrid (Spain) and the Department of Biology of Organisms and Systems (BOS) of the University of Oviedo (Spain), were examined. In the “Material Examined” section of the species description, all localities with collection data and additional information are fully listed. All reported species are based upon verified records by the authors, through the evaluation of direct field results and preserved physical evidence that exhibits diagnostic features. Additional information used to assess the diversity of medicinal leeches in Spain was obtained from scientific published data [1,2,3,4,5,6,7,8,9,10,11,12,13,14,15,16,17,18,19,20,21,22,23,24,25,26,27,28,29,30,31,32,33,34,35,36,37,38,39,40,41,42,43,44,45].

Overall morphology and anatomy were examined under both dissecting stereomicroscope and compound light microscope. Selected specimens were dissected dorsally and ventrally for anatomical study. Line drawings were made with the aid of a camera lucida and digital photography. Photomicrographs were taken with a Leica DFC310FX camera mounted on a Leica M205FA stereomicroscope.

To define the specific or subspecific identity of the studied taxa, we used an integrative approach including tree topologies, pairwise uncorrected p–distance, Automatic Barcoding Gap Discovery (ABGD), and morphological and anatomical data. Measurements and counts in the descriptions are of the holotype; the range for the paratypes is given in parentheses; terminology of general morphology follows [1,3,36]. The specimens examined in this study are deposited in the MNCN of Madrid and the “Grigore Antipa” National Museum of Natural History of Bucharest, Romania (MGAB).

### 2.2. Molecular Methods

Ten 100% ethanol fixed specimens of Iberian *Hirudo* from the two morphotypes (“typical” and “bilineated”) collected from five Spanish localities (Bikuña, Cueza, Gándaras de Budiño, Doniños, and Ocelo; see Figure 1 and Table 1) were prepared for genetic analysis. The specimens collected for molecular analysis were deposited in the Annelida Collection of the “Grigore Antipa” National Museum of Natural History of Bucharest (Romania, MGAB), under the following inventory numbers: ANN003-ANN012. Tissue samples were taken from the caudal sucker to avoid contamination from gut contents. Approximately half of the caudal sucker was removed with a scalpel and the tissue fragments were processed thereafter. Genomic DNA was extracted using the Isolate II Genomic DNA Kit (Bioline, London, UK) following manufacturer specifications. A partial COI fragment was amplified using the universal PCR primers LCO1490 and HCO2198 [37]. The mitochondrial 12S rRNA gene and the nuclear Internal Transcribed Spacer (ITS2) gene were amplified with primers developed by [1] and references therein.

The PCR reactions were performed in a total volume of 50 μL containing 10 ng of DNA template, 1X Green GoTaq^®^ Flexi Buffer, 2.5 mM MgCl2, each dNTP at 0.1 mM, 0.5 μM of each primer and 1.5 units of GoTaq^®^ DNA polymerase (Promega, Madison, WI, USA). The PCR products were isolated from samples presenting clean and visible bands on 0.5 μg ml-1 ethidium bromide stained agarose gel, using the FavorPrep™ Gel/PCR Purification Kit (FAVORGEN^®^ Biotech Corp., Changzhi, Taiwan), following manufacturer specifications. Macrogen (Seoul, Korea) services were used for sequencing.

The resulting sequences for both genes were edited and aligned in CodonCode Aligner version 3.7.1. (CodonCode Corporation, Dedham, MA, USA). For this study, another 45 sequences of COI and 12S for all studied species of *Hirudo*, as well as 28 sequences of the Internal Transcribed Spacer (ITS2) of *H. verbana* and *H. troctina*, and one outgroup, were retrieved from GenBank (Table A1).

The unique haplotypes in the whole data set were identified in DnaSP v.5 [38] and were used for all the subsequent analysis. Genetic distances between groups (either recognized species or morphotypes of *H. verbana*) were calculated in MEGA7 [39]. A median-joining network was constructed in PopART v. 1.7. [40], with haplotypes represented by circles with sizes proportional to the number of individuals, while different colours were used to depict the geographic distribution of the haplotypes.

In order to evaluate the phylogenetic relationships between Iberian *Hirudo* and its related species, we performed a phylogenetic analysis by Bayesian inference using MrBayes v3.2.6 [41] on the platform CIPRES Science Gateway (https://www.phylo.org accessed on 15 June 2020). First, we used PartitionFinder v.2 [42] to identify the optimum partition scheme and substitution models for the concatenated sequences and preliminary exploratory analyses were performed for each gene fragment. As the resulting tree topologies did not contradict each other, we decided to perform the further analysis on the combined COI and 12S mitochondrial sequences. Based on the proposed partitions/models, we used one partition for COI codon 1 and 3 and 12S, with the K81UF + I + G model of molecular evolution, while a second partition was used for COI codon 2 with the K81UF+G model. We ran two simultaneous analyses with ten million generations each, sampling every 1000th generation. Gaps were treated like missing data and default settings were used for all other parameters.

Consensus trees obtained by summarizing the posterior sample of trees to produce a maximum clade credibility tree with default burn-in value generated using TREEANNOTATOR [43]. FIGTREE v1.4.4 [44] was used for visualising trees and producing publication-quality figures.

The Automatic Barcoding Gap Discovery (ABGD) [45] analysis was used for molecular species delimitation as implemented on the web server http://wwwabi.snv.jussieu.fr/public/abgd/abgdweb.html (accessed on 18 June 2020). A sequence alignment was submitted and the analysis was performed on the Jukes-Cantor and Kimura 2-P genetic distances matrix, with the relative gap width between 0.5 and 1.5, and a prior intraspecific divergence (from Pmin to Pmax) between 0.1% to 10%, with 10 steps.

## 3. Results

### 3.1. Phylogenetic Reconstruction

**Figure 2:**
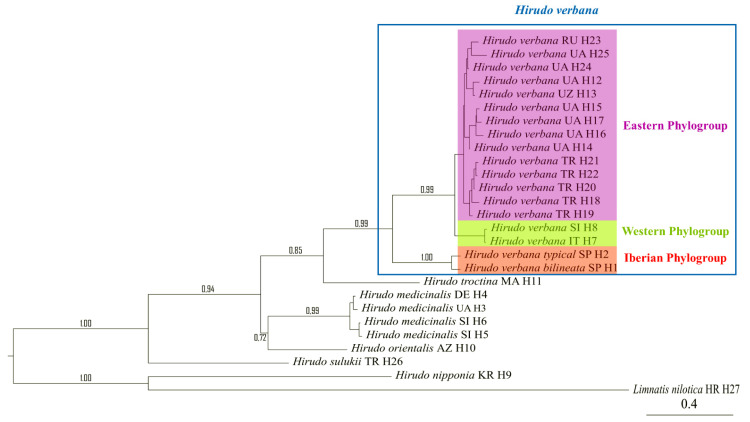
Phylogenetic relationship of *Hirudo* spp. based on Bayesian inference for the combined COI and 12S mitochondrial markers.

Final alignments of the concatenated 55 sequences of the two analysed mitochondrial markers, COI (606 bp) and 12S (286 bp) rRNA, revealed 27 unique haplotypes of the treated *Hirudo* spp. Two new haplotypes of COI and 12S rRNA (“H 1” and “H 2”) have been identified in the *Hirudo* analysed samples from Spain. “H 1” was found in all specimens of the “bilineated” morphotype from the Basque Country and Léon, and “H 2” in all specimens of the “typical” form from Galicia (Table 1). We identified nine parsimony informative sites in the two haplotypes, six of which were found in the COI portion of the combined sequences (all mutated positions were producing differences in the amino-acid sequence), while only three mutated positions were found in the 12S portion of the combined sequences. The Bayesian Analysis of the two concatenated mitochondrial genes supports the monophyly of the group formed by the two Iberian morphotypes of *Hirudo*, which are sister to *H. verbana* (Figure 2). Our analyses confirmed the two previously reported and well-supported clades of *H. verbana* (i.e., the Eastern and Western Phylogroups *sensu*, Trontelj and Utevsky [4]) and evidenced the existence of a new phylogroup, which is herein designated as the “Iberian phylogroup” (Figure 2). The new Iberian phylogroup of *H. verbana* is composed of all collected specimens from Spain (Figure 1). The internal resolution of this phylogroup, comprising the “bilineated” and the “typical” morphotypes, was robustly supported in our analysis (BS = 99–100%).

**Figure 3:**
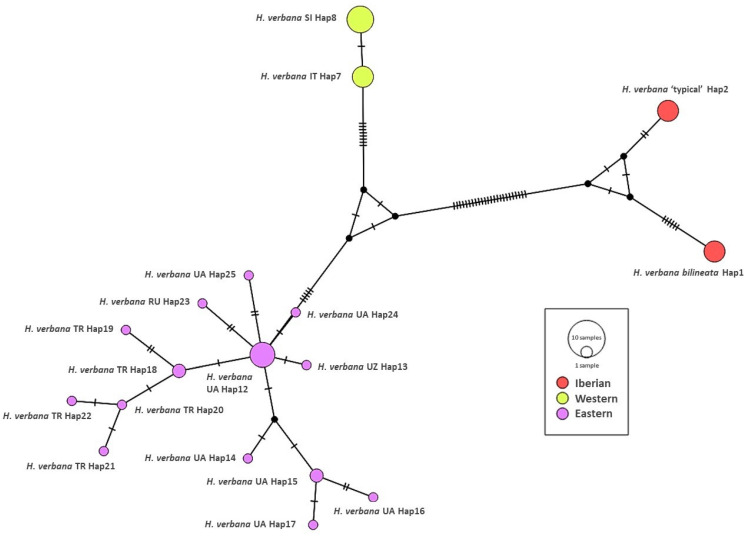
Median joining network of *Hirudo verbana.* The bars on branch length represent the number of substitutions.

The Median-Joining network showing the relationships among haplotypes of *H. verbana* is depicted in Figure 3. It can be observed that the nucleotide differences are limited to one or two mutational positions between different haplotypes in each lineage. For the Eastern *H. verbana* lineage, we obtained a dominant haplotype H 12, found in individuals from south-western, southern, and north-eastern Ukraine, the Krasnodar, and Stavropol territories of the Russian Federation, one of the most important centres of commercial harvesting with medicinal leeches, where the releasing of animals after their use may change the intraspecific genetic structure. The star-like shape of the network with a rarer haplotype radiating from the common haplotype obtained for the Eastern lineage suggests a fast range expansion from a relatively small founding population. The Western lineage is represented by two haplotypes, which differ by only one mutated position. The new Iberian phylogroup comprises two haplotypes with nine mutations between them (see Figure 3).

The calculated genetic distances between groups using the p-distance method are presented in Table 2. The distances between Iberian “bilineated” and “typical” morphotypes of *H. verbana* and the two Eastern and Western phylogroups of the same species are between 0.039 and 0.043. The genetic distance between the two Spanish morphotypes (1%) is not significantly different from the genetic distance between the Eastern and Western phylogroups of *H. verbana* (1.80%), considering the standard error. The pairwise genetic distances computed between all the other analysed species ranged from 7.30% (*H. orientalis*—*H. medicinalis*) to 10.30% (*H. sulukii*—*H. troctina*) and to 20.80% (*H. nipponia*—*H. orientalis*), and were significantly higher than the distances between the *H. verbana* groups.

The Automatic Barcoding Gap Discovery (ABGD) analysis correctly identified all used species when the prior intraspecific divergence varied between 2.20% and 3.60%. When this prior value was as low as 1.30%, the analysis identified the Iberian clade as a distinct group from all the other *H. verbana* samples. Finally, at 0.70% intra-specific divergence, the analysis identified the Western and Eastern lineages of *H. verbana*, as well as the “bilineated” and “typical” morpho-groups from Spain. The same result (groups separating at the same levels of intraspecific genetic divergence) was obtained when using Jukes-Cantor or Kimura 2-P distances and for values of the relative gap width between 0.5 and 1.5.

The ABGD analyses revealed the existence of cryptic diversity within the studied samples of *H. verbana*. These analyses recovered the same four groups as in the phylogenetic analyses. Thus, *H. verbana* is shown to be formed by a complex of four phylogroups (Eastern Group, Western Group, Iberian “typical”, and Iberian “bilineated”). Morphologically, based on the knowledge we have to date, the first three are virtually indistinguishable (i.e., “cryptic”) and are considered as belonging to the nominal morpho-subspecies (*H. verbana verbana*), while the last one can be clearly differentiated from the other three and thus is herein described as a new subspecies.

### 3.2. Taxonomic Account

Systematic position according to Tessler et al. [23].

Phylum Annelida Lamarck, 1809

Class Clitellata Michaelsen, 1919

Subclass Hirudinea Lamarck, 1818

Order Hirudinida Siddall et al., 2001

Suborder Hirudiniformes Caballero, 1952

Genus *Hirudo* Linnaeus, 1758

Species *Hirudo verbana* Carena, 1820

*Hirudo verbana bilineata* ssp. nov.

Type material: Holotype: One adult individual preserved in ethanol, 56 mm long, 10.5 mm wide, Bikuña pond, 855 m altitude, 42°50′16″ N, 2°19′24″ W, Bikuña (San Millán), Álava, Basque Country, Spain (MNCN 16.02/123).

Paratypes: Six adult specimens preserved in ethanol, ranging from 42.5 to 76 mm in length, and from 6 to 9.5 mm in width, same data as holotype (MNCN 16.02/124).

Non-type material: Three adult specimens, same data as holotype; 12 adult specimens from Cueza pond, 913 m altitude, 42°25′35″ N, 4°55′56″ W, Celada de Cea, León, Spain (BOS Collection; MNCN); 4 specimens, Zuazo de Cuartango, Kuartango Valley, 555–1000 m altitude, Álava, Basque Country, Spain, Coll. F.J. Ocharan 1980 (BOS Invertebrate Collection, University of Oviedo).

Comparative material: *Hirudo verbana* “typical”: Location data and other important information is fully listed below in the section of “*Hirudo verbana verbana* Iberian typical”.

**Figure 4:**
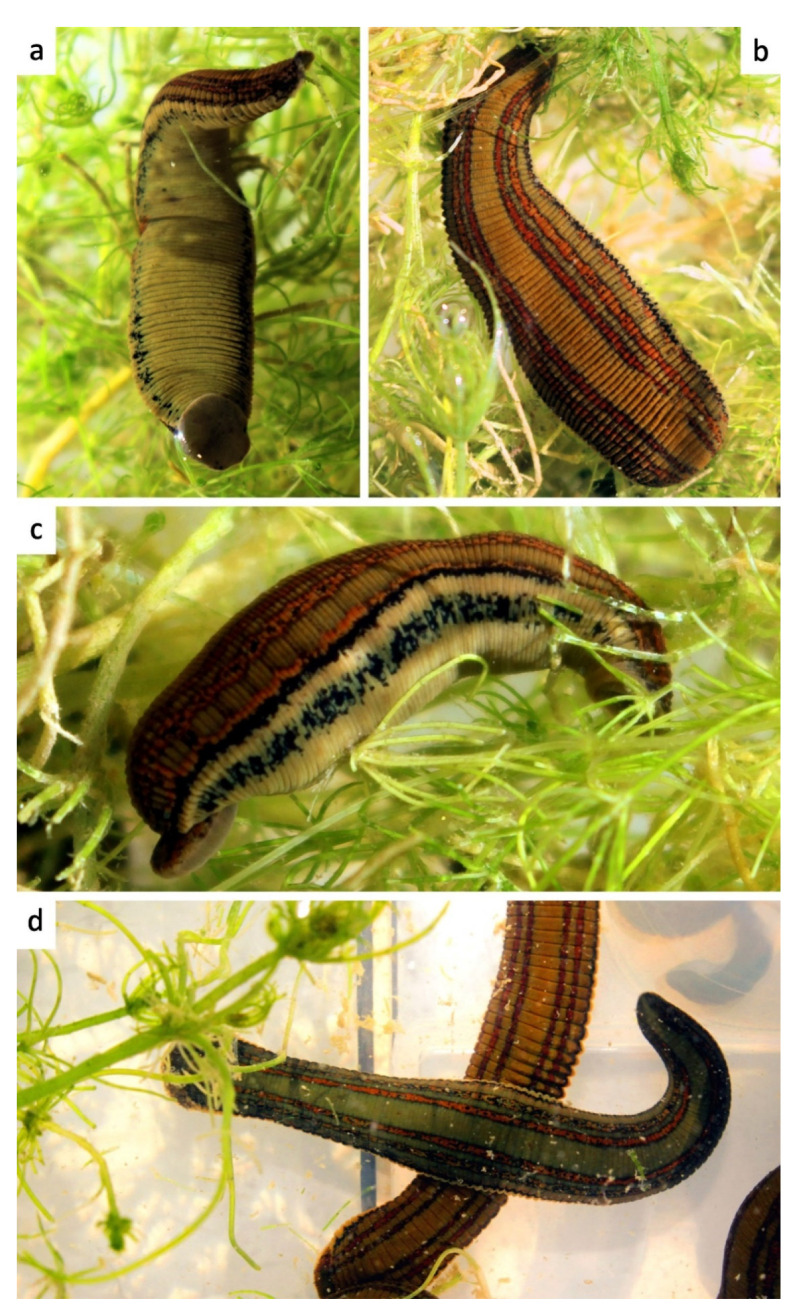
*Habitus* and colour pattern of living specimens of *Hirudo verbana bilineata* ssp. nov. from the type locality. (**a**,**b**) Ventral and dorsal views of holotype; (**c**) lateral view of topotype specimen; (**d**) topotype specimens with greenish background-colour (above) and with brownish one (below).

**Figure 5:**
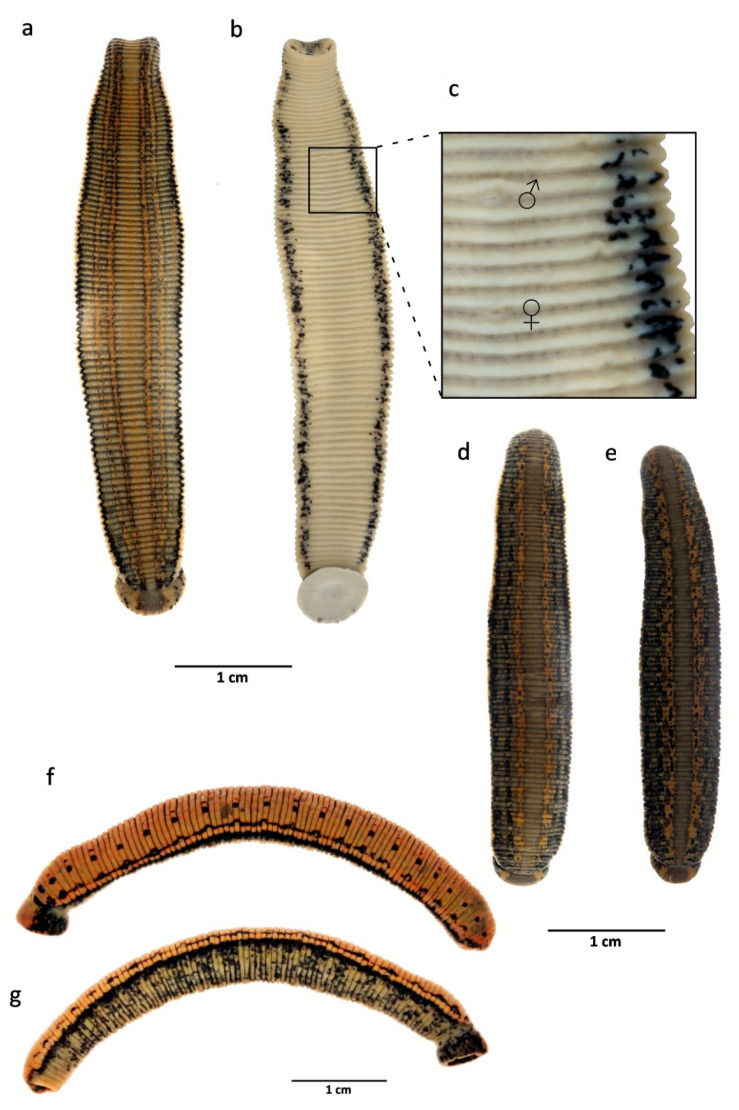
Iberian species of medicinal leeches treated in this work. (**a**,**b**) dorsal and ventral views of the holotype of *Hirudo verbana bilineata* ssp. nov; (**c**) detailed view of genital pores of the same; (**d**,**e**) *Hirudo verbana* “typical” from Galicia (NW Spain), dorsal view; (**f**,**g**) dorso-lateral and ventro-lateral views of *Hirudo troctina* from Asturias (N Spain).

Etymology: The subspecific name is derived from the Latin meaning “with two lines”, with reference to the dorsal colour pattern consisting of two thin, paired lines that run parallel on both sides of dorsum along its whole length (Figure 4 and Figure 5).

Diagnosis: Medium-sized species, maximal length of about 80 mm including suckers; pigmentation of dorsum brownish to greenish with two pairs of distinct orange/reddish longitudinal lines or stripes, with or without small quadrangular or rounded dark spots on stripes; lateral margins with a longitudinal beige/light yellow stripe; venter unicoloured greenish to yellowish, without coloured markings and bounded by a pair of black ventrolateral stripes; jaws trignathous, monostichodont, papillated; no pharyngeal ridges terminating between jaws; epididymides medium-sized; vagina centrally swelled and curved.

Description: External characters: Length up to 80 mm after fixation, maximum body width 10.5 mm, width of anterior sucker 5 mm (2.5–5), width of posterior sucker 7.5 mm (4–8); gonopores separated by five annuli, male pore in the furrow XI b5/b6, female pore in the furrow XII b5/b6; complete segment five-annulated (b1, b2, a2, b5, b6); body surface covered with numerous papillae; five pairs of eyes, on II, III, IV a1, V a1, and VI a2, in a horse-shoe arrangement; sulcus present as a narrow, distinct groove running from the crypt of the median dorsal jaw to the dorsal rim of the anterior sucker; dorsum brownish or greenish with two pairs of narrow orange or reddish longitudinal stripes (being sometimes both colour present on same individual: external line orange and inner one reddish) edged with black, with or without small quadrangular, rounded, or irregular dark spots on stripes (Figure 4a–c and Figure 5a,b); if present, marginal stripes yellow, edged with black line; venter unicoloured greenish, with two irregular black ventrolateral stripes (Figure 4a and Figure 5b,c).

Mouthparts: Jaws trignathous, monostichodont, papillated.

**Figure 6:**
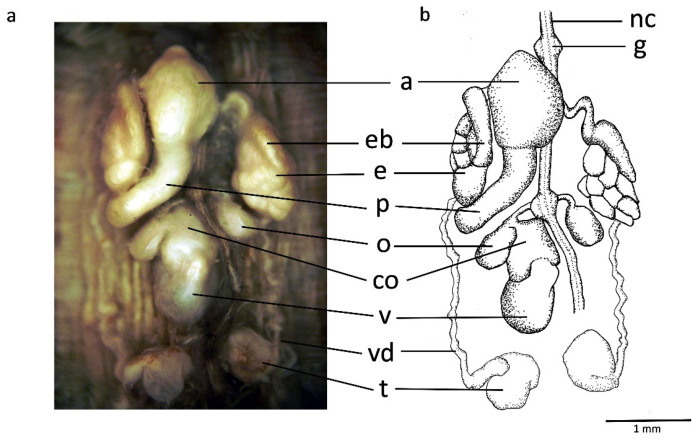
*Hirudo verbana bilineata* ssp. nov. (**a**,**b**) dorsal view of reproductive system of nov. a, atrium, co, common oviduct, e, epididymis, eb, ejaculatory bulb, g, ganglion in segment XI, nc, ventral nerve cord, o, ovisac, p, penis sheath, t, testisac, v, vagina, vd, vas deferens.

Male reproductive system: Large and bulging atrium covered by a glandular layer and ubicated at ganglion in segment XI; penis sheath as long broad duct anteriorly bent and reaching ganglion in segment XII. Discoid and medium-sized epididymides, as tightly packed masses of ducts standing upright on either side of atrium and located between ganglia in segments XI and XII. Fusiform and well-developed ejaculatory bulbs and not larger than epididymides, the dorsocefalic faces of which they circle. Thin vasa deferentia running from epididymides to posterior end. Nine pairs of testisacs present; two anteriormost ones rounded, approximately 1.5 times larger than ovisacs and located posterior to ganglion in segment XIII (Figure 6).

Female reproductive system: Vagina as upright, centrally swelled, curved tube entering directly in ventral body wall, posterior to ganglion in XII. Common oviduct reaches vagina subterminally at a small vaginal caecum. Common oviduct as thin duct with several loops and covered by a thick glandular layer bound to the cephalic face of vagina. Globular and small ovisacs. Whole female reproductive system located between ganglia XII and XIII (Figure 6).

**Figure 7:**
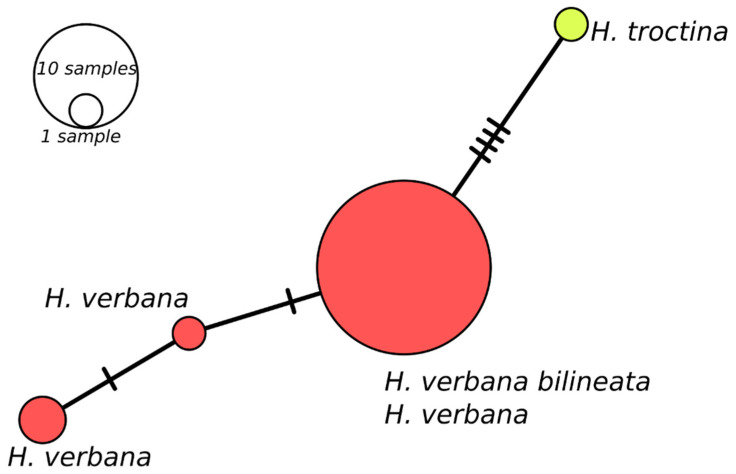
Minimum spanning network of *Hirudo verbana* and *Hirudo troctina* ITS2 haplotypes. The bars on branch length represent the number of substitutions.

Remarks: *Hirudo verbana bilineata* is very similar to other *H. verbana* in a number of external and internal characters; however, it can be easily distinguished from them using some manifest features. The pigmentation is the most helpful character in identifying these leeches, *H. verbana bilineata* differs from the remaining forms of *H. verbana* by bearing two thin, paired, and deep orange to reddish lines that run parallel on both sides of the dorsum along its whole length, whereas the other *H. verbana* morphs have broad and diffuse paramedian stripes which are pale orange in colour (this pattern is referred here as “typical”). The venter of all known forms of *H. verbana*, including *H. verbana bilineata*, is unicolored greenish to yellow, bounded by a pair of black ventrolateral stripes, while *H. medicinalis* and *H. orientalis* present a dark ventral pigmentation, varying from an irregular mesh-like pattern in the former to a more regular, with segmentally arranged pairs of light markings on a black background in *H. orientalis*. In northern Spain, *H. verbana bilineata* occurs sympatrically with *H. troctina*. However, these species tend to occupy different habitats (this statement is more detailed explored in the discussion below). Both species can be easily distinguished by the dorsal and ventrolateral colour patterns that are strictly different. *Hirudo troctina* has a distinctive pair of zigzag-shaped and black ventrolateral longitudinal stripes, which is absent in all other European *Hirudo* species. For the sake of completeness, an alternative and putative scenario has been assessed. This is that the new subspecies could be a hybrid of *H. verbana* and *H. troctina*. The first observation that rules out this “hybridization scenario” is the fact the two supposedly parent species exhibit different habitat preference and therefore they cannot interact under natural conditions (i.e., there is an ecological barrier between both species). We also addressed this scenario from a genetic point of view. If the hybridization occurred, *H. verbana* should have acted as a female (since all our putative hybrid specimens share the same mtDNA with *H. verbana*) and *H. troctina* as a male. Thus, the presumed hybrids would be the offspring of a female *H. verbana* and a male *H. troctina*. The analysis of a nuclear gene, which exhibits differences between *H. verbana* and *H. troctina,* should allow us to recover (in the supposed hybrids) the paternal (*H. troctina*) gene variant. Therefore, we sequenced the Internal Transcribed Spacer (ITS2) nuclear marker in four specimens of the *H. verbana bilineata* and compared them with 27 ITS2 sequences of *H. verbana* and one of *H. troctina* retrieved from Genbank [4]. This revealed that all *H. verbana bilineata* specimens belong to a single haplotype (GenBank acc. no. MW820085), which is also the most frequent haplotype found among *H. verbana*. The only found *H. troctina* ITS2 haplotype is easily set apart from the *H. verbana* haplotypes, as depicted by a Minimum Spanning Analysis implemented in PopART v. 1.7. [40] (Figure 7). Thus, from both an ecological and genetical point of view, the hybridization scenario is highly unlikely, and therefore, it can be objectively discarded.

**Figure 8:**
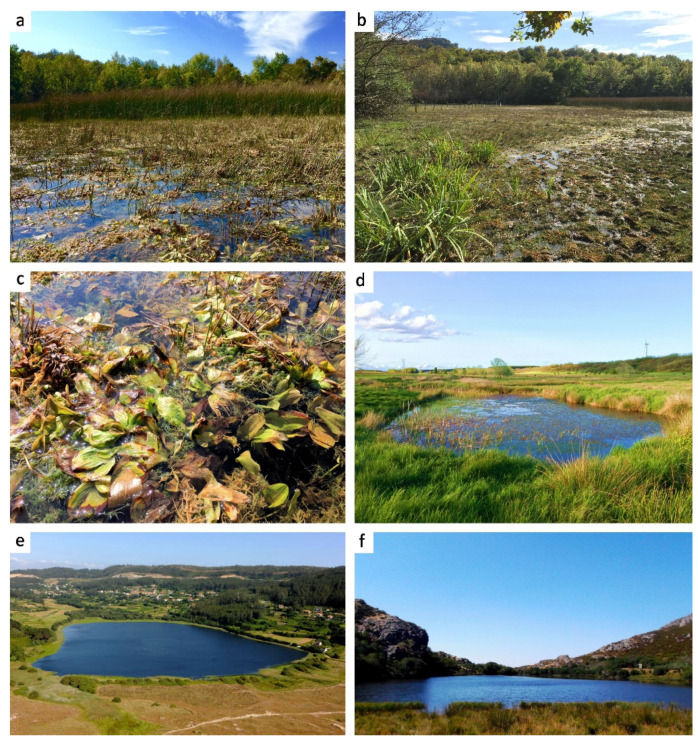
Habitats of *Hirudo verbana* in Spain. (**a**,**b**) Bikuña pond (Basque Country), type locality of *H. verbana bilineata* ssp. nov.; (**c**) detailed view of the same showing the *Chara-Potamogeton* association; (**d**) Cueza pond (León); (**e**) Doniños Lake (Galicia); (**f**) Ocelo lake (Galicia).

Habitat and distribution: *Hirudo verbana bilineata* is only known from the standing waters of hills and mountain plains pools of the Basque Country and León (northern Spain) (Figure 1 and Figure 8). Current populations of this new subspecies (Bikuña and Cueza) are located on an ecotone or “transition area” between the Atlantic Cornisa Cantábrica (Atlantic Eurosiberian Biogeographic Region) and the Mediterranean Valley of Ebro River (Mediterranean Biogeographic Region) (Figure 1). The examination of historical collections of the BOS Department (University of Oviedo) demonstrated that this subspecies was also present in several ponds from the Kuartango Valley (500–1000 m altitude, Basque Country, N Spain) until the 90s, when the leeches became extinct from the area [31]. No *H. verbana* specimens were found in the Collections of the MNCN, all *Hirudo* specimens belong to *H. troctina*. The latter species was also present in the BOS Collection from some mountain streams of Asturias (northern Spain) (Figure 5f,g). It is important to note that all examined specimens of *H. troctina* were collected from moving water bodies, such as rivers and streams, while all *H. verbana* specimens found came from habitats that are primarily standing waters, such as lakes and ponds.

This new subspecies was found in clear, still, lentic, or low-flowing waters of 0.1–1.5 m depth, as part of the *Chara-Potamogeton* community. *Potamogeton* sp. is a profusely branched, rhizomatous perennial with submerged filiform leaves (Figure 8c), which constitutes a sheltered three-dimensional habitat to a great number of associated animals. The Iberian green frog (*Pelophylax perezi*) and the Alpine newt (*Ichthyosaura alpestris*) were very common in the *H. verbana bilineata* habitat and both amphibians were observed acting as hosts of this new subspecies in nature.

In relation to the physicochemical parameters, the Bikuña pond presents a marked seasonality. In the summer months it suffers from low-water but does not get completely dry. Its maximum depth does not exceed 1 m. The water temperature varies from 4 °C in the winter months (never reaching the freezing point) to over 20 °C, reaching temperatures above 25 °C on the hottest days of the summer. The pH through the whole annual cycle is always above 7, normally varying in a range between 7.5 and 8.5 (Red de seguimiento del estado ecológico de los humedales interiores, Agencia Vasca del Agua [https://www.uragentzia.euskadi.eus/red-de-seguimiento-del-estado-ecologico-de-los-humedales-interiores-20162017/u81-0003771/es/] accessed on 29 June 2020).

*Hirudo verbana* cf. *verbana* Carena, 1820 (“Iberian typical”)

Examined material: *Hirudo verbana* “typical”: Two specimens, Doniños lake, 4.5 m altitude, 42°29′31″ N, 8°18′44″ W, A Coruña, Galicia, Spain; two specimens, Gándaras de Budiño lake, 20 m altitude, 42°06′43″ N, 8°37′48″ W, Pontevedra, Galicia, Spain; one specimen, Water channel of Antela lake, Antela Valley, 610 m altitude, 42°05′24″ N, 7°45′00″ W, Ourense, Galicia, Spain; four specimens, Xuño lake, 4 m altitude, 42°38′00″ N, 9°02′18″ W, A Coruña, Galicia, Spain; three specimens, Ocelo lake, Pena Trevinca mountains, 1517 m altitude, 42°13′36″ N, 6°52′22″ W, Ourense, Galicia, Spain. For historical examined material see Table 3.

Diagnosis: Medium-sized species, maximal length of about 70 mm including suckers; maximum body width 9 mm; pigmentation of dorsum greenish with an orange-pigmented longitudinal reticulum; lateral margins with a longitudinal beige to yellow stripe; venter unicoloured greenish to yellowish, with no (or very few) dark spots and a pair of black marginal stripes (Figure 5d,e); jaws trignathous, monostichodont, papillated; no pharyngeal ridges terminating between jaws; epididymides medium-sized; vagina centrally swelled and curved.

Habitat and distribution: *Hirudo verbana* cf. *verbana* is only known from the lakes, ponds, and pools of Galicia and the Basque Country from 4 to 1517 m of altitude (NW to N Spain) (Figure 1 and Figure 8; Table 3). However, in our sampling, this subspecies was only found in Galicia. The Basque Country populations registered from the historical collections are most likely extinct today [31].

*Hirudo troctina* Johnson, 1816

Diagnosis: Small to medium-sized species, medium length of about 50 mm including suckers; pigmentation of dorsum greenish to greyish with four or six dorsal metameric black dots; lateral margins with a longitudinal orange stripe; venter unicoloured greenish to yellowish, with dark spots and a pair of black marginal stripes arranged in zigzag (Figure 5f,g); jaws trignathous, monostichodont, papillated; with six muscular ridges fusing in pairs to form three, each of which terminates at the base of a jaw; epididymides very large; broad, elongate, and centrally swelled vagina and tapering at both ends.

Examined material: See Table 3.

Habitat and distribution: High to medium flow rivers and streams from southern, central and northern Spain. Our last confirmed historical record of the species dates back 1992 from northern Spain (Table 3). No specimens were found in our sampling from 2015 to 2017.

## 4. Discussion

Medicinal leeches were historically popularised for their utility in bloodletting (phlebotomy) in the 18th and 19th centuries. They have experienced a recent prominence again in post-operative treatments for flap and replantation surgeries, and in terms of characterization and isolation of salivary anticoagulants [1,6,24]. Medicinal leeches were raised for commercial purposes in ponds in the 18th and 19th centuries from several European countries, including Spain and its neighbours Portugal and France. Millions of these leeches were used in hospitals, barber shops, and pharmacies of the time [25,26]. There was a huge trade in medicinal leeches, and local production was commonly supplemented by importations from abroad [26,27]. Only in Spain, the import/export traffic by maritime trade in the mid-19th century, accounted for between 10,000 and 1 million individuals per year [26]. This triggered a massive over-exploitation of natural populations of medicinal leeches, causing these species across some areas, like northern Spain, to became scarce since the mid-19th century [13,15]. Despite their great past and present importance in medicine and pharmacology, our knowledge of medicinal leech diversity, distribution, and zoogeography is still incomplete. A clear example of this is represented by the Iberian medicinal leeches, which have been studied insufficiently and unsystematically. Here, we report for the first time the occurrence of *H. verbana* from the Iberian Peninsula, constituting its westernmost distribution to date.

Iberian populations correspond to two different morphotypes, which are also supported by molecular data. It is widely accepted that external pigmentation is not only one of the most useful features to distinguish leech species, but also arguably the best character to diagnose species within the genus *Hirudo* [1,3,6,24]. Apart from the smaller size of the Iberian *H. verbana* specimens with “typical” colour (max. 70 mm long/9 mm width) in relation to the other European clades (max. 100–140 mm/10–12 mm width) [1,3,6,24], we did not find other clear enough morphological differences and thus we do not formally describe them as a subspecies in the present study. However, we found genetic differentiation in the analysed mitochondrial markers and therefore, we referred to it as *H. verbana* cf. *verbana*. These findings suggest that the genetic diversity within *H. verbana* is considerably higher than it was accepted before. Thus, each of the four provided clades within *H. verbana* may represent a subspecific status. This hypothesis should be investigated further based on examination of additional material and genetic markers. The taxonomic rank of subspecies remains highly contentious, largely because traditional subspecies boundaries have sometimes been contradicted by molecular data [28]. However, our results demonstrate a high level of congruence between “morphosubspecies” and molecular phylogenetic data. The distribution range of Iberian *Hirudo* morphotypes is divided into two well-delimited geographical groups with some morphological differences. *Hirudo verbana bilineata* inhabits the mountain small ponds or pools (of more than 800 m high) of the Cantabrian Mountains (“Cordillera Cantábrica” in Spanish) that stretch for over 300 km across northern Spain (Figure 1 and Figure 8a–c). Otherwise, *H. verbana* “typical” was found from large lakes at lower altitude from the Galician Massif in Galicia (NW Spain) (Figure 1 and Figure 8e,f). Using an integrative approach (a combination of morphology and molecular data), we found that the two geographical groups should be considered as two separate taxa. Accordingly, we formally describe the new subspecies *H. verbana bilineata*. Morphological analyses support the differentiation of this new subspecies from the other Iberian morphotype and the remaining Iberian phylogroups of *H. verbana*. There are strong diagnostic characters to differentiate them based upon the colour pattern and size. In addition to morphology and genetics, slightly divergent habitat preferences and the disjunctive distribution of the two subspecies also support the recognition of the new taxa. Iberian *H. verbana* populations (i.e., the Iberian phylogroup) constitute an important component in the evolutionary legacy of the European medicinal leeches, and thus, the populations of *H. verbana bilineata* and “typical” *H. verbana*, are here considered as Evolutionarily Significant Units (ESUs). Accordingly, they should be treated as distinct and as a priority for purposes of conservation. This is a pivotal point, because Iberian medicinal leech populations have been declining in recent decades as both direct and indirect results of human activity [13,15,17,31].

Some northern Iberian literature records of *H. medicinalis* and *H. officinalis* may actually refer to this new subspecies [18,29]. However, most of them do not provide information about their colour patterns nor photographs or drawings of the specimens, so they cannot be formally confirmed. As an exception, Laborda [30] in his handbook of the fauna of León presents a photograph of a live specimen of presumed *H. medicinalis* showing the typical bilineated pattern of *H. verbana bilineata* [30]. In the same way, the illustrated specimens reported as *H. medicinalis* by Ocharan [31] from Kuartango (Alava, Basque Country) and by García-Mas and Muñoz [15], are indeed *H. verbana* of the “typical” pattern [15,31]. Similarly, it is likely that Portuguese medicinal leeches also belong to *H. verbana*, since they were traditionally marketed as “green leeches” or “Lisbon brand” (common names attributed to *H. verbana*) [28]. Conversely, the leeches from northern Europe (*H. medicinalis*) were historically known as “Black leeches” or “Hamburg brand” [32]. Ayres and Comesaña-Iglesias [16] have reported *H. medicinalis* as being a parasite on the Iberian brown frog *Rana iberica*. Although it is difficult to ascertain from the non-diagnostic photograph provided, the location where they found this medicinal leech (i.e., a small stream in Barranqueira de Casariños, Pontevedra, Galicia) falls within the distribution area of the “typical” morph of *H. verbana* herein reported. Therefore, these leeches may be referred to as *H. verbana*. On the contrary, in other records of amphibian predation by *H. medicinalis* from northern Spain [33], it is evident (thanks to the provided images) that the species was misidentified and does not correspond to any member of the genus *Hirudo*, but most likely to the horse-leech *Haemopis sanguisuga*.

Blanchard [18] noted that *H. troctina* was not confined to southern Spain as previously considered. Almost 100 years later, Fernández-Bernaldo-de-Quirós [34] reported the species from two Asturian rivers near their mouths in the Cantabrian sea (between 0.5 and 10 m altitude) [34]. The conducted revision, based upon historical collections (Table 3), confirms the occurrence of this species in northern Spain, specifically from A Coruña and Lugo and as far as 43° lat. N (Asturias). Furthermore, our results have shown that *H. troctina* differs in its habitats from *H. verbana,* preferring moving bodies of water to lentic ecosystems. This statement is also supported by bibliographic data, since, to our knowledge, all the Iberian records come from rivers or streams (see [18,19,20,34,35]).

Furthermore, there are no reports of co-occurrence of these two species in the same water body, so it is unlikely that both species can outcompete each other. Taking into account the new provided information on the distribution and morphological diversity of *H. verbana* in Iberia, the previously reported *Hirudo* spp. records should be taken with caution until new evidence can confirm that these populations are still extant.

It is very likely that before Pleistocenic glaciations *H. verbana* occurred throughout the Western Palaearctic. The major clades of *H. verbana* (the Western and Eastern phylogroups and the Iberian superclade), which do not overlap in their geographical distribution, support the hypothesis of a distinct postglacial colonization process from Mediterranean peninsulas [4]. The Eastern phylogroup recolonized vast territories of the eastern steppe and arid landscapes, while Western and Iberian phylogroups are restricted to the Apennine and Iberian peninsulas and adjacent areas. Moreover, the geographic structuring of the two Iberian lineages of medicinal leeches (*H. verbana bilineata* in the upper reaches of the Duero, Ebro and northern rivers catchment and *H. verbana* “typical” in the Miño-Sil watershed) indicates survival in at least two different Pleistocene glacial *refugia*. Therefore, the Iberian Peninsula is regarded more likely as a cradle for freshwater endemisms rather than a single *refugium*, as has been previously suggested by Gómez and Lunt [11]. Certainly, Iberian *H. verbana* ssp. distribution patterns contribute to a better understanding of the internal complexity of the Iberian Peninsula as a source of glacial relics/endemic taxa, harbouring populations with a high degree of genetic structure that began to settle throughout the Pleistocene.

## 5. Conclusions

Iberian *Hirudo* populations are declining in recent decades as both a direct and indirect result of human activity. We have here described *H. verbana bilineata*, which was only found in two localities in low numbers. Thus, this new subspecies, as well as the typical morph of *H. verbana*, can be considered as locally rare taxa, being also consistent with the definition of an Evolutionarily Significant Unit. Rarity of species is usually explained by a combination of extensive versus restricted geographic range, broad versus narrow habitat tolerance and large versus small population size. Iberian *H. verbana* populations combine restricted geographic ranges with narrow habitat and small population size, making them vulnerable to extinction. Therefore, there is an urgent need to assess in detail the conservation status and to initiate conservation measures for this endemic subspecies and its congeners, in order to reverse their decline and recover them.

Despite the long history of studies about the taxonomy and phylogeny of medicinal leeches, our knowledge is still incomplete. Undoubtedly, further investigations involving testing of nuclear genetic markers and morphological variation in “Iberian typical” *H. verbana* is required. Application of scanning electron microscopy and molecular tools may shed light on the evolutionary history of medicinal leech populations from other Mediterranean peninsulas and the Western European countries, which may preserve unrecognized diversity of *Hirudo* species. The broader view is that *H. verbana* subspecies/phylogroups can provide an effective short-cut for estimating patterns of intraspecific genetic diversity, thus constituting a useful tool for the study of evolutionary divergence and conservation in medicinal leeches in Europe.

## Figures and Tables

**Table 1:** Location of studied Spanish samples used in this analysis with combined COI and 12S mt markers.

Sample ID	Species	Morphotype	Locality	Collector /Date
ANN003	*Hirudo verbana*	“bilineated”	Bikuña pond, 42°50′16″ N, 2°19′24″ W, Álava, Basque Country	A. Arias 26 September 2015
ANN004	*H. verbana*	“bilineated”	Bikuña pond, 42°50′16″ N, 2°19′24″ W, Álava, Basque Country	A. Arias 26 September 2015
ANN005	*H. verbana*	“bilineated”	Bikuña pond, 42°50′16″ N, 2°19′24″ W, Álava, Basque Country	A. Arias 26 September 2015
ANN006	*H. verbana*	“bilineated”	Bikuña pond, 42°50′16″ N, 2°19′24″ W, Álava, Basque Country	A. Arias 26 September 2015
ANN007	*H. verbana*.	“bilineated”	Cueza pond, 42°25′35″ N, 4°55′56″ W, León, Castilla y León	R. Carballeira 22 May 2016
ANN008	*H. verbana*	“typical”	Gándaras de Budiño, 42°06′43″ N, 8°37′48″ W, Pontevedra, Galicia	R. Carballeira 13 June 2015
ANN009	*H. verbana*	“typical”	Gándaras de Budiño, 42°06′43″ N, 8°37′48″ W, Pontevedra, Galicia	R. Carballeira 13 June 2015
ANN010	*H. verbana*	“typical”	Doniños Lake, 42°29′31″ N, 8°18′44″ W, A Coruña, Galicia	R. Carballeira 13 June 2015
ANN011	*H. verbana*	“typical”	Doniños Lake, 42°29′31″ N, 8°18′44″ W, A Coruña, Galicia Spain	R. Carballeira 13 June 2015
ANN012	*H. verbana*	“typical”	Ocelo, 42°13′36″ N, 6°52′22″ W, Orense, Galicia	R. Carballeira 13 June 2015

**Table 2:** The p-distances calculated between groups of *Hirudo* based on haplotypes of concatenated COI and 12S sequences. Standard error estimate(s) are shown above the diagonal.

	1	2	3	4	5	6	7	8	9	10
1 *H. medicinalis*		1.3%	0.9%	0.9%	0.9%	0.9%	0.9%	0.9%	0.9%	1.4%
2 *H. nipponia*	20%		1.4%	1.3%	1.3%	1.3%	1.3%	1.3%	1.3%	1.4%
3 *H. orientalis*	7.3%	20.8%		0.9%	0.9%	0.9%	0.9%	0.9%	1%	1.4%
4 *H. troctina*	8%	18.9%	8.4%		0.9%	0.9%	0.9%	0.9%	1%	1.4%
5 *H. verbana* Eastern Phylogroup	8.3%	19.5%	8.1%	8.6%		0.4%	0.6%	0.6%	1%	1.4%
6 *H. verbana* Western Phylogroup	7.7%	18.8%	7.6%	7.7%	1.8%		0.7%	0.7%	1%	1.4%
7 *H. verbana verbana* (Iberian)	7.5%	18.8%	7.5%	7.6%	3.9%	4.1%		0.3%	1%	1.4%
8 *H. verbana bilineata*	7.7%	19.6%	7.6%	7.9%	4.3%	4.3%	1%		1%	1.4%
9 *H. sulukii*	9.4%	19.7%	9.6%	10.3%	10%	9.8%	9.7%	10%		1.4%
10 *Limnatis* cf. *nilotica*	23.1%	24.7%	22.9%	22.9%	23.7%	23.4%	23.2%	23.4%	22.5%	

**Table 3:** Summary of historical collection *Hirudo* specimens examined in this study.

Species	No. of Specimens	Habitat	Locality	Coll. Date	Reg. Number
*Hirudo troctina* Johnson, 1816	6	Niebla stream	Plasencia, Cáceres, Extremadura	May 1944	MNCN 16.02/85
*Hirudo troctina* Johnson, 1816	1	Alagón river	Coria, Cáceres, Extremadura	May 1944	MNCN 16.02/84
*Hirudo troctina* Johnson, 1816	3	Tiétar river	Casavieja, Ávila, Castilla y León	May 1932	MNCN 16.02/81
*Hirudo troctina* Johnson, 1816	1	Jerte river	Plasencia, Cáceres, Extremadura	May 1944	MNCN 16.02/71
*Hirudo troctina* Johnson, 1816	1	Arrago river	Moraleja, Cáceres, Extremadura	June 1944	MNCN 16.02/70
*Hirudo troctina* Johnson, 1816	1	Panes stream	Panes, Peñamellera Baja, Asturias	August 1992	BOS Collection
*Hirudo troctina* Johnson, 1816	1	Panes stream	Panes, Peñamellera Baja, Asturias	August 1992	Utevsky S. Collection
*Hirudo troctina* Johnson, 1816	1	Bedón river	Naves, Llanes, Asturias	1980s	BOS Collection
*Hirudo troctina* Johnson, 1817	1	Pas river	Vioño de Piélagos, Piélagos, Cantabria	1980s	BOS Collection
*Hirudo verbana* cf. *verbana* Carena, 1820	1	Kuartango Valley ponds	Zuazo de Cuartango, Álava, Basque Country	1980-1990	Utevsky S. Collection
*Hirudo verbana* cf. *verbana* Carena, 1820	20	Kuartango Valley ponds	Zuazo de Cuartango, Álava, Basque Country	1980-1990	BOS Collection

## Data Availability

All data generated or analysed during this study are included in this published article. Type material is deposited in the Museo Nacional de Ciencias Naturales (MNCN) of Madrid, Spain. The new subspecies is deposited in ZooBank with accession number urn:lsid:zoobank.org:act:7A75F602-2BF7-4686-BF9B-C83CBCDBC94C. All sequences were deposited in GenBank (https://www.ncbi.nlm.nih.gov/nuccore/ accessed on 17 June 2020) with accession number from MT797288 to MT797292 (COI) and from MT796846 to MT796850 (12S).

## Appendix A

**Table A1:** Analysed samples with accession numbers, codes, haplotypes and reference.

Species/Provenance	GenBank Acc. No COI	GenBank Acc. No 12S	Haplotype	Reference
*Limnatis* cf. *nilotica*	AY763152	AY763161	H27	(Trontelj & Utevsky [1])
*Hirudo medicinalis* Linnaeus, 1758				
Gorelaya Dolina, Kharkiv Region, UA	AY763148	AY763156	H3	(Trontelj & Utevsky [1])
Saale River, Rotherburg, Halle, DE	AY763148	AY763157	H4	(Trontelj & Utevsky [1])
Podvinci pond, Ptuj, SI	AY763149	AY763158	H5	(Trontelj & Utevsky [1])
Petajnci gravel pit; Murska Sobota, SI	AY763149	AY763159	H6	(Trontelj & Utevsky [1])
*Hirudo verbana* Carena, 1820				
Lecce, IT	AY763150	AY763160	H7	(Trontelj & Utevsky [1]))
Lake Ohrid, Ohrid, MK	AY763150	AY763160	H7	(Trontelj & Utevsky [1])
Pond Globobaj near Povir, Seqana, SI	AY763151	AY763160	H8	(Trontelj & Utevsky [1])
PA55, Island Pag, HR	EF446690	AY763160	H8	(Siddall et al. [5])
PA54, Island Pag, HR	EF446691	AY763160	H8	(Siddall et al. [5])
OH33, Lake Ohrid, MK	EF446693	AY763160	H7	(Siddall et al. [5])
OH31, Lake Ohrid, MK	EF446694	AY763160	H7	(Siddall et al. [5])
KR41, Gabrovica, SW, Sl	EF446695	AY763160	H8	(Siddall et al. [5])
KR40, Gabrovica, SW, Sl	EF446696	AY763160	H8	(Siddall et al. [5])
X1, Lecce, S IT	EF446698	AY763160	H7	(Siddall et al. [5])
KA49 Povir, SW SI	EF446699	AY763160	H8	(Siddall et al. [5])
KA47 Povir, SW SI	EF446700	AY763160	H8	(Siddall et al. [5])
KA46 Povir, SW SI	EF446701	AY763160	H8	(Siddall et al. [5])
Kharkiv Reg., Horila Dolyna, NE, UA	JN083793	JN104650	H12	(Siddall et al. [5])
Berezivka, SW, UA	JN083793	JN104650	H12	(Siddall et al. [5])
Karateri, UZ	JN083793	JN104652	H13	(Siddall et al. [5])
Kokhany, S, UA	JN083793	JN104655	H12	(Siddall et al. [5])
Krasnodar, RU	JN083793	JN104649	H12	(Trontelj & Utevsky [4])
Severynivka, SW, UA	JN083793	JN104650	H12	(Trontelj & Utevsky [4])
Stavropol, RU	JN083793	JN104650	H12	(Trontelj & Utevsky [4])
Vilkove, SW, UA	JN083793	JN104649	H12	(Trontelj & Utevsky [4])
CRIMEEA. UA	JN083794	JN104654	H14	(Trontelj & Utevsky [4])
Berezivka, SW, UA	JN083795	JN104650	H15	(Trontelj & Utevsky [4])
Shaby, SW, UA	JN083795	JN104649	H15	(Trontelj & Utevsky [4])
Mayaky, Odessa Region, SW, UA	JN083796	JN104653	H16	(Trontelj & Utevsky [4])
Severynivka, SW, UA	JN083798	JN104651	H17	(Trontelj & Utevsky [4])
Izmir, TR	JN083800	JN104649	H18	(Trontelj & Utevsky [4])
North_TR	JN083800	JN104649	H18	(Trontelj & Utevsky [4])
Izmir, TR	JN083801	JN104649	H19	(Trontelj & Utevsky [4])
HV11, Izmir, TR	JN083802	JN104649	H20	(Trontelj & Utevsky [4])
HV12, Izmir, TR	JN083803	JN104649	H21	(Trontelj & Utevsky [4])
HV13, Izmir, TR	JN083804	JN104649	H22	(Trontelj & Utevsky [4])
HV14, Stavropol, RU	JN104641	JN104650	H23	(Trontelj & Utevsky [4])
HV15, Berezivka, SW, UA	JN104642	JN104650	H24	(Trontelj & Utevsky [4])
HV17, MalaK, S, UA	JN104644	JN104649	H25	(Trontelj & Utevsky [4])
ANN003, Bikuña pond, Bikuña, Cuadrilla de Salvatierra, Álava, Basque Country, SP	MT797288	MT796846	H1	Present study
ANN004, Bikuña pond, Bikuña, Cuadrilla de Salvatierra, Álava, Basque Country, SP	MT797288	MT796846	H1	Present study
ANN005, Bikuña pond, Bikuña, Cuadrilla de Salvatierra, Álava, Basque Country, SP	MT797288	MT796846	H1	Present study
ANN006, Bikuña pond, Bikuña, Cuadrilla de Salvatierra, Álava, Basque Country, SP	MT797288	MT796846	H1	Present study
ANN007, León, Cueza, Castilla y León, SP	MT797289	MT796847	H1	Present study
ANN008, Galicia BUD 3, Gándaras de Budiño pond Pontevedra, Galicia, SP	MT797290	MT796848	H2	Present study
ANN009, Galicia BUD 4 Gándaras de Budiño pond Pontevedra, Galicia, SP	MT797290	MT796848	H2	Present study
ANN010, Galicia DON 3 Doniños Lake, A Coruña, Galicia, SP	MT797291	MT796849	H2	Present study
ANN011, Galicia DON 4 Doniños Lake, A Coruña, Galicia, SP	MT797291	MT796849	H2	Present study
ANN012, Galicia OCE 2 Orense, Galicia, SP	MT797292	MT796850	H2	Present study
*Hirudo nipponia* Whitman, 1886				
Leech farm Hans Biomed, KR	AY763153	AY763162	H9	(Trontelj & Utevsky [1])
*Hirudo orientalis* Utevsky & Trontelj, 2005				
Unknown locality in Azerbaijan, AZ	AY763154	AY763163	H10	(Trontelj & Utevsky [1])
Agdam District, AZ	AY763154	AY768704	H10	(Trontelj & Utevsky [1])
*Hirudo troctina* Johnson, 1816				
Bazaar in Marrakech, MA	AY763155	AY763164	H11	(Trontelj & Utevsky [1])
*Hirudo sulukii* Saglam, Saunders, Lang and Shain 2016				
HS1, Kara Lake, Adiyaman, TR	KU216239	KU216262	H26	(Saglam et al. [7])
